# Unmeasurable Stimulated Thyroglobulin Before Radioactive Iodine Ablation Predicts Excellent Long-Term Outcomes in Patients with Differentiated Thyroid Cancer

**DOI:** 10.3390/cancers18071058

**Published:** 2026-03-25

**Authors:** Yi Sia, Radu Mihai

**Affiliations:** Department of Endocrine Surgery, Churchill Cancer Centre, Oxford University Hospitals NHS Foundation Trust, Oxford OX3 7LE, UK; huanyi.sia@nh.org.au

**Keywords:** thyroid cancer, thyroglobulin, recurrence

## Abstract

***Background***: There is ongoing interest in limiting the extent of treatment for patients with low-risk differentiated thyroid cancer (DTC) and in redesigning individualised follow-up strategies. This study assessed long-term outcomes in patients with excellent response to surgical treatment demonstrated by reaching unmeasurable stimulated thyroglobulin (sTG) levels before proceeding with radioactive iodine ablation. ***Methods***: This is a retrospective cohort study of consecutive patients treated for DTC in a tertiary referral centre. Radioactive iodine ablation (RIA) was done after hormone withdrawal (before 2015) or after Thyrogen stimulation (in recent years). The biochemical assay for TG changed from a lower limit of detectability of 5 ng/mL to 0.2 ng/mL in 2012. ***Results***: Of 331 patients operated on between 2001 and 2019, unmeasurable sTG was measured in 70 of 138 patients (51%) when using an assay with threshold of 5 ng/mL and in 38 of 193 patients (20%) based on the threshold of 0.2 ng/mL. Compared with patients whose sTG was >5 ng/mL, those with sTG <5 ng/mL (187 of 331 patients) were less likely to have T3–T4 tumours or positive lymph node disease (N1a–N1b) and had a lower MACIS score (5.73 ± 1.26 vs. 6.45 ± 1.69, *p* < 0.001) and much lower mortality with metastatic disease during follow-up for 100 ± 48 months (3/187 vs. 23/144 patients, *p* = 0.001). ***Conclusions***: Patients with unmeasurable sTG have excellent prognosis, with very low incidence of adverse events. With the wide use of TG assay with a threshold of 0.2 ng/mL (or lower), this subgroup could have patient-initiated follow-up rather than embark on regular assessments.

## Simple Summary

Following treatment for thyroid cancer, measuring of thyroglobulin (TG) levels is an important parameter to assess the response to treatment and to estimate the risk of tumour recurrence. Measuring stimulated TG (sTG) in the presence of high TSH levels before proceeding with radioactive iodine ablation is expected to provide additional information about the effectiveness of surgical treatment. This study used a cohort of 331 patients who had thyroidectomy and radioactive iodine ablation stratified based on sTG values reached before proceeding with radioactive iodine ablation. It was found that during a median follow-up of over 8 years, patients with unmeasurable sTG have very low risk of recurrence or mortality. A proposal is made that such patients could be discharged from long-term follow-up.

## 1. Introduction

It is expected that thyroid cancer will become the third-most common cancer [1] as a result of its increasing incidence, which has tripled over the last four decades up to the current level of approximately 14 per 100,000 individuals [2]. Some called this phenomenon an ‘epidemic of diagnosis not disease’ [3], and concerns regarding this trend were discussed in an editorial as an example of overdiagnosing diseases with minimal clinical significance [4]. Because many thyroid cancers are part of a reservoir of non-fatal tumours that are increasingly being overdetected [5], it becomes imperative to establish risk-stratified treatment strategies in order to avoid overtreatment and excessive prolonged follow-up assessments.

Despite the increasing incidence of differentiated thyroid cancer (DTC) over the past five decades, the mortality from thyroid cancer has remained low and not significantly changed [6,7,8]. This increase in diagnosis of DTC is largely made up of small cancers (i.e., those measuring 2 cm or smaller) [9] and has been attributed to the increased sensitivity of ultrasound scans and the wider use of cross-sectional imaging [8]. Guidelines have been updated to help mitigate overdiagnosis and prevent overtreatment. The American Thyroid Association revised its three-tier risk stratification system in 2015, which consists of low-, intermediate-, and high-risk groups for estimating the likelihood of developing persistent or recurrent disease and emphasised that the risk of disease recurrence is a continuum ranging from <1% in very low-risk patients to >50% in high-risk patients [10]. While this risk stratification system helps guide treatment pathways, it provides minimal details for individualised follow-up strategies according to the risk of recurrence.

Long-term prognosis of DTC is very good, with an overall survival rate at 10 years of 80–95% [11], but recurrence rates as high as 35% have been reported after 40 years of follow-up [12]. This makes the number of patients who might require surveillance for disease recurrence considerable, placing added strain on any healthcare system. While stratified pathways for patient-initiated follow-ups have been discussed or established for several types of cancer [13,14,15], no such protocols exist for thyroid cancer. In this context, survival is an impractical parameter to be monitored in any studies assessing the impact of the care pathways on the long-term outcomes. For this reason, monitoring the incidence of local or distant recurrence based on radiological or biochemical assessment is more informative.

Thyroglobulin (TG) is an iodinated glycoprotein which is normally stored in the follicular colloid (a substrate for thyroid hormone production) of the thyroid [16]. Because TG is secreted only by thyroid cells, TG serves as a specific and accurate tumour marker in patients with thyroid cancer who undergo total thyroidectomy and radioactive iodine ablation (RIA), with the expectation that effective treatment leads to very low or undetectable levels of TG and that a rise in TG would signal disease recurrence. Measuring TG levels during thyroxine replacement (i.e., when TSH is suppressed) is part of follow-up protocols after treatment for thyroid cancer. In addition, stimulated thyroglobulin (sTG) measured in the presence of high TSH induced by Thyrogen administration or by withdrawal of thyroid hormone replacement prior to RIA has emerged as an independent predictor of clinical outcomes in DTC patients [17,18,19,20,21,22,23,24]. Low pre-ablation sTG values have a high negative predictive value, making them a sensitive tool for stratifying DTC-related risk [22,24,25]. It remains unclear if sTG could be used as a marker of quality of surgical excision, with the expectation that incomplete resections would lead to a higher sTG in the first few weeks after surgical treatment and before proceeding with RIA.

The aim of this study was to characterise clinical outcomes in a large cohort of patients with DTC, with emphasis on the correlation between pre-ablation sTG levels and long-term outcomes.

## 2. Materials and Methods

### 2.1. Patient Selection

A retrospective cohort study of consecutive patients treated for DTC in the last two decades was undertaken in a tertiary referral centre. Only patients who received RIA after total thyroidectomy or after lobectomy followed by completion thyroidectomy were included. Data on patients’ demographics and clinical, operative, biochemical, and radiological details were collected from hospital electronic patients records and historical records. Patients with missing information on their pre-ablation sTG or TSH values, those with TSH < 20 mU/L on the day of RIA, and those who had poorly differentiated or anaplastic tumours were excluded. The study was registered as a local audit of outcomes in patients with thyroid cancer (10754/2025).

### 2.2. Thyroglobulin Assay

Until September 2012, TG assays were performed at the Queen Elizabeth Hospital, Birmingham, UK, using a radioimmunoassay with a detection limit of 5 ng/mL. Between September 2012 and June 2019, TG was measured in-house using the Siemens thyroglobulin immunoassay (Immulite 2000, Siemens, UK), with a detection limit of 0.2 ng/mL. This was subsequently switched to the Architect i2000 immunoassay (Abbott Diagnostics, UK) in June 2019 but without changes in the detection limit of 0.2 ng/mL. Pre-ablation stimulated thyroglobulin was performed on the morning of the admission for radioactive iodine ablation, which corresponded to day 3 of Thyrogen treatment for the recent patients.

Thyroglobulin antibodies (TgAbs) were measured predominantly in the last decade. Patients who did not have TgAbs measured, or those with elevated TgAbs above the upper limit of normal (115 IU/mL), were nonetheless included in our analysis due to the variability of antibody assays during the study period [26].

### 2.3. Extent of Surgery

Patients with DTC diagnosed on either fine-needle aspirate (FNA) or incidentally after thyroidectomy were discussed at the regional multidisciplinary team (MDT) meeting, and each patient’s treatment was tailored accordingly. During the study, there was a change in local policy, moving from the initial policy of offering total thyroidectomy and RIA to all tumours > 1 cm to the more recent policy of restricting this treatment to tumours > 4 cm or those presenting with locally advanced disease (such as those with cervical lymphadenopathy).

All operations were performed by a consultant endocrine surgeon as the main operator or as supervisor of a postgraduate fellow or advanced trainee with a dedicated interest in thyroid surgery. Thyroidectomies were performed in each of the four hospitals within the Thames Valley thyroid cancer network. All patients undergoing lateral lymph node dissection were operated on in the tertiary referral centre by one of two designated consultants. As part of the local cancer network arrangements, all surgeons operating on patients with thyroid cancer maintained a minimum annual workload of 20 thyroid cases per year as recommended by the British Association of Endocrine and Thyroid Surgeons guidelines.

MACIS score was calculated using the online Thyroid Cancer Staging Calculator (AJCC 8th Edition) (accessed at https://www.thyroid.org/professionals/calculators/thyroid-cancer-staging-calculator/). Post-ablation iodine uptake in the cervical area was calculated as a percentage of the initial dose administered. These values were calculated at the time of treatment and collected retrospectively from information available on electronic patient records.

### 2.4. Radioactive Iodine Ablation Protocol

During postoperative discussion in the multidisciplinary meeting, the indication for RIA was decided in line with current guidelines. According to historical changes in local protocol, RIA was performed either after withdrawal of liothyronine for 14 days or after administration of Thyrogen. I^131^ was administered if TSH > 30 mU/L, and at that time point, sTG was measured. This homogenous approach over nearly two decades makes the comparison of outcomes over such a long time period reasonable. The standard dose of I^131^ was 3.1 GBq, and occasionally, 5.1 GBq was used if deemed beneficial.

### 2.5. Outcomes

Disease-free status was allocated to all patients whose post-RIA scans showed no evidence of extra cervical disease and whose TG during follow-up remained unmeasurable (<0.2 ng/mL). Disease recurrence was demonstrated on biochemical criteria (i.e., based on raising TG levels) with or without radiological evidence for the site/extent of recurrence based on cervical ultrasound assessment and CT images, as deemed clinically indicated at the time of their assessments.

### 2.6. Data Analysis

Statistical analysis was performed using StatPlus (AnalystSoft Inc.—a statistical analysis program for macOS Version v8 (see https://www.analystsoft.com/en/). Normally distributed data are reported as mean ± std; otherwise, the median (interquartile range) was presented. Nonparametric values were compared using Mann–Whitney U test for independent variables. A *p*-value of <0.05 was deemed significant.

## 3. Results

### 3.1. Patient Cohort

Between January 2001 and September 2019, 447 consecutive adult patients were treated with RIA. Their records were scrutinised, and 116 patients were excluded from further analysis if pre-ablation sTG values were not measured (n = 73), if final histological analysis reported that the tumour exhibited features of poorly differentiated (n = 4) or anaplastic carcinoma (n = 2), if histological data regarding the primary tumour was not available (n = 21), if the indication for thyroidectomy was to facilitate radioactive iodine ablation after diagnosis of malignant struma ovari (n = 1), or if follow-up data were shorter than 2 years (n = 15).

**Figure 1:**
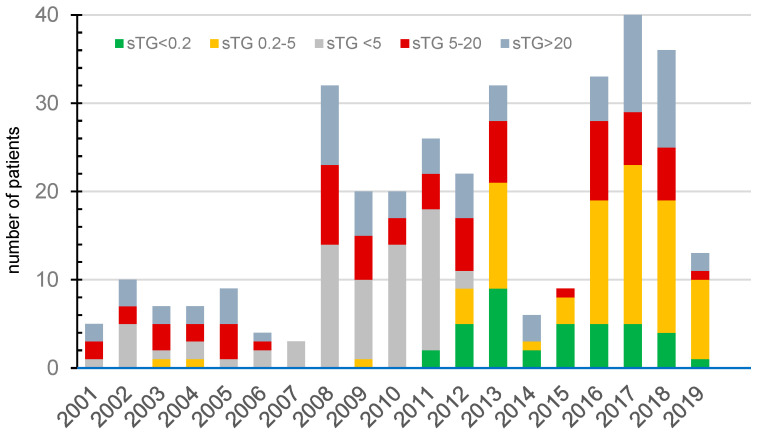
Yearly recruitment of patients into the study as stratified based on the pre-ablation stimulated thyroglobulin levels.

The yearly recruitment into the study is illustrated in Figure 1. The lower number of patients in the first decade was related to the fact that measuring pre-ablation sTG was not part of routine practice before 2008. Over two-thirds of patients were operated on in the tertiary referral centre—the hub of the multidisciplinary network (229 of 331 patients, 69%).

**Figure 2:**
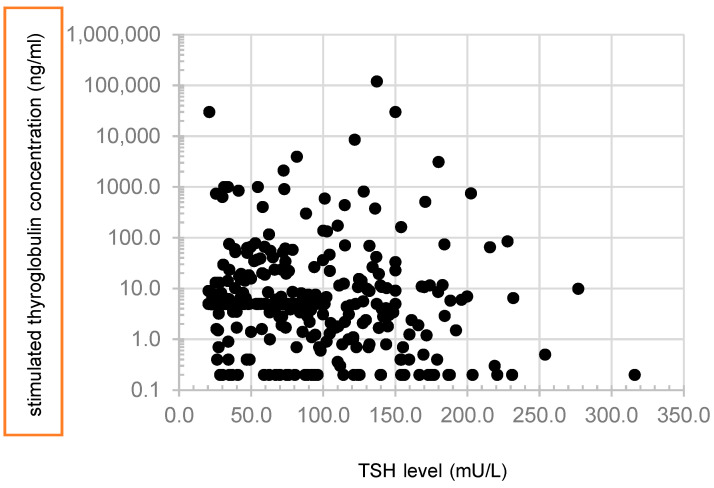
Individual values of stimulated thyroglobulin and TSH levels before radioactive iodine ablation in a cohort of 331 patients.

Patients included in this analysis were predominantly females (230F:101M). The average age was 50 ± 17 years, with significantly lower value in female patients (48 ± 17 vs. 54 ± 17 years, *p*= 0.009). At the time of the ablation, median TSH level was 75.7 mU/L (IQR 40.5–125.9). Individual values for TSH and sTG are illustrated in Figure 2.

**Figure 3:**
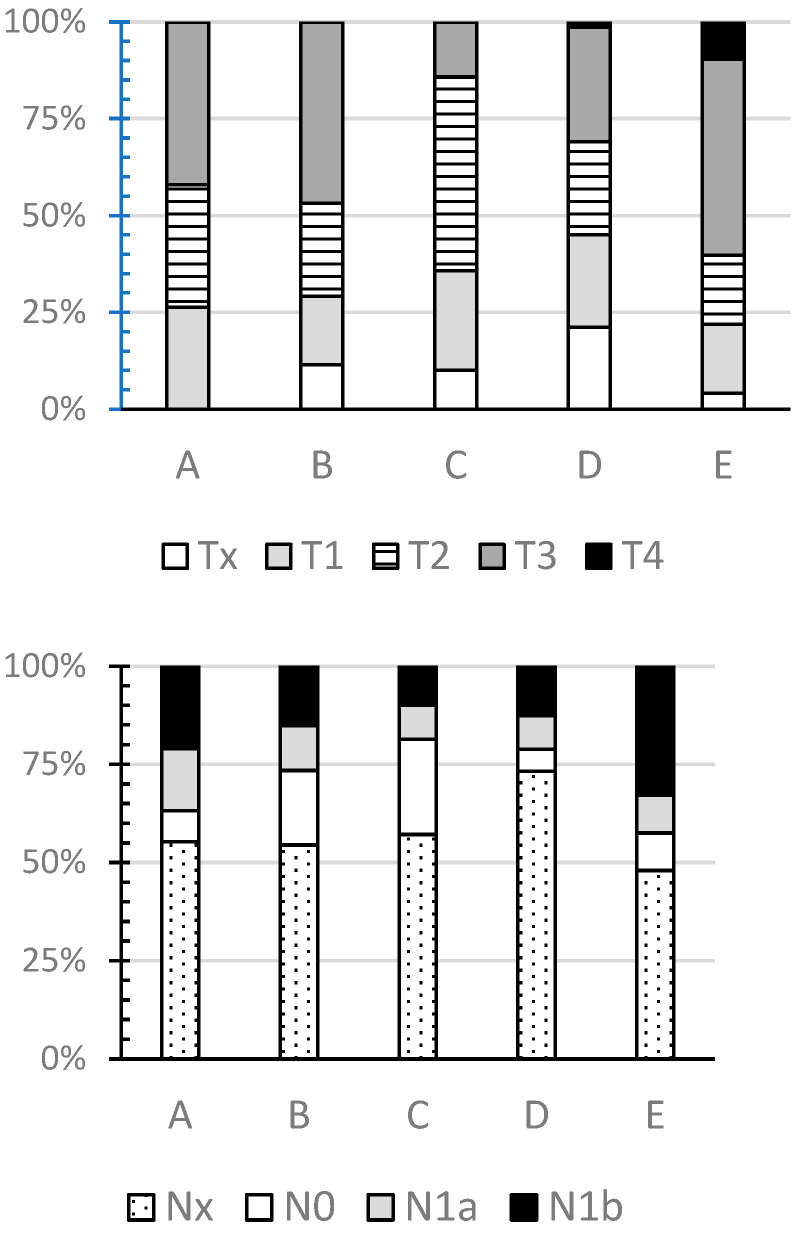
Distribution of T- and N-stages between subgroup of patients stratified based on the pre-ablation stimulated thyroglobulin levels.

The entire cohort of 331 patients was divided in five subgroups based on sTG values: group A—sTG unmeasurable defined as <0.2 ng/mL (n = 38); group B—sTG 0.2–5 ng/mL (n = 79); group C—sTG unmeasurable defined as <5 ng/mL (n = 70); group D—sTG 5–20 ng/mL (n = 71); and group E—sTG > 20 ng/mL (n = 73). The value of 20 ng/mL used to separate groups D and E was chosen based on the median value of sTG in patients with values over 5 ng/mL (i.e., above the level considered unmeasurable based on historical TG assay). The clinical and pathological details of patients in each of these subgroups are summarised in Table 1. The gender distribution and age of patients in the five subgroups were similar (*p* = NS). The distribution of TNM stages in each subgroup is illustrated in Figure 3, demonstrating that surgery for T4 tumours led to pre-ablation sTG > 20. Out of 60 patients with N1b disease, 15 patients (25%) were found to have unmeasurable sTG (either at the threshold of <5 ng/mL (n = 7) or with the newer threshold of 0.2 ng/mL (n = 8)), and a further 12 patients had sTG 0.2–5 ng/mL, suggesting that presentation with lateral cervical lymphadenopathy (N1b) disease did not preclude achievement of unmeasurable/low sTG.

Before the change in TG assay in 2012, unmeasurable sTG (<5 ng/mL) was recorded in 70 of 138 patients (51%). After the change in TG assay, unmeasurable sTG, defined as <0.2 ng/mL, was recorded in 38 of 193 patients (20%), and values <5 ng/mL were measured in 117 of 193 patients (60%). In the entire cohort, a total of 187 patients had sTG less than 5 ng/mL (combined subgroups A–B–C), and they were less likely to have tumours classified as T3–T4 (63/187 vs. 66/144, chi2 test 0.0003) or positive lymph node disease N1a–N1b (48/187 vs. 46/144, chi2 test 0.0002), and had lower MACIS score (5.73 ± 1.26 vs. 6.45 ± 1.69, *p* < 0.001), compared with patients whose sTG was greater than 5 ng/mL (combined subgroups D–E, n = 144). In the entire study group, a similar proportion of patients achieved sTG less than 5 ng/mL when operated on in the tertiary centre (132 of 229 patients, 57%) or in the regional district hospitals (47 of 102 patients, 54%).

sTG < 1 ng/mL was measured in 66 patients, of whom 62 patients (94%) had no evidence of disease after a follow-up of 84 ± 30 months, two patients had biochemical recurrence (46 and 52 months, respectively), and two patients died of unrelated causes at 25 and 163 months after initial surgery for thyroid cancer.

Anti-thyroglobulin antibodies were raised in 16 patients equally distributed between the five subgroups of patients. Most of these patients had antibodies titres checked in the most recent years (i.e., values were not known at the time of their initial treatment), and therefore, initial clinical decisions were not affected by this information.

### 3.2. Cervical Iodine Uptake Between Subgroups

**Figure 4:**
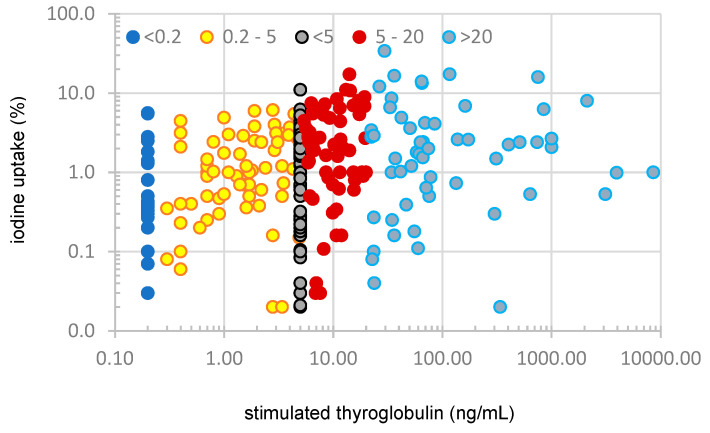
Individual values of cervical radioactive iodine uptake after I^131^ ablation in a cohort of 331 patients.

RIA was performed at a median of 12 weeks after thyroidectomy. Cervical iodine uptake in individual patients, calculated as percentage of the dose administered, is illustrated in Figure 4, and the median values for each subgroup are detailed in Table 2. There was no correlation between sTG and the cervical uptake of 131I, but patients with sTG less than 5 ng/mL (subgroups A–B–C) had lower cervical uptake (0.9% (IQR 0.3–2.2%)) compared with those with sTG greater than 5 ng/mL (subgroups D–E) whose median uptake was 1.9% (IQR 0.6–4.5%) (*p* = 0.001).

### 3.3. Long-Term Outcome of Patients Stratified Based on Pre-Ablation sTG

Clinical status was assessed at a mean of 100 ± 48 months after initial surgical treatment. Overall results are summarised in Table 3, and the main observations were as follows:

Group A. All but one patient had no evidence of disease at the time of the last assessment. Only one patient died of unrelated cause.

Group B. The vast majority of patients were deemed to have no evidence of disease (70/79, 89%), and six patients had biochemical evidence of disease (median TG, 10.6 ng/mL). Two patients died of unrelated causes, and a single patient died with metastatic disease.

Group C. Most of the patients (56/70, 80%) had no evidence of disease, four had biochemical recurrence (median TG 2.2 ng/mL), and two patients had structural recurrence. Two patients died of metastatic disease, and six other patients died of unrelated causes.

Group D. Biochemical recurrence (n = 8; median TG, 1.05 ng/mL) and structural recurrence (n = 1) became apparent early in the course of disease in this subgroup. Two patients died of metastatic thyroid cancer, and six patients died of unrelated causes.

Group E. Only a minority of patients (16/73, 22%) were disease-free, while many had biochemical and/or radiological evidence of recurrent disease (31/73, 43%). All but three deaths (21 of 24) in this subgroup were related to metastatic thyroid cancer.

Overall, death in the presence of metastatic disease was recorded in 3 of 187 patients whose pre-ablation sTG was less than 5 ng/mL and in 23 of 144 patients with higher sTG (*p* = 0.001). Patients who died of metastatic thyroid cancer were slightly older at the time of initial diagnosis compared with the overall cohort (68 ± 13 vs. 50 ± 17 years, *p* = 0.04), and both genders were equally distributed (23F:21M). All patients with pre-ablation sTG < 20 ng/mL survived in excess of 10 years while those with sTG > 20 ng/mL had a median survival of only 37 months (IQR 24–97 months).

**Figure 5:**
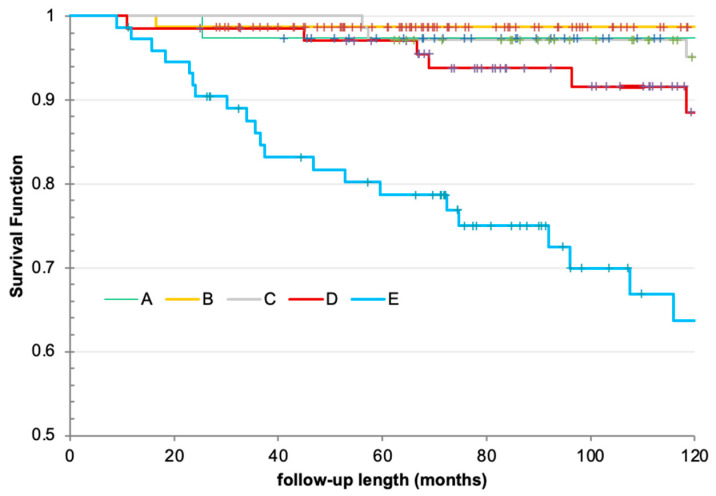
Kaplan–Meier survival curves for patients stratified based on sTG before proceeding with radioactive iodine ablation.

Differences in overall survival between subgroups are illustrated in Figure 5. Worse survival associated with sTG > 20 ng/mL had a hazard ratio (HR) of 11.6 when compared with patients with sTG < 0.2 ng/mL and HR 4.2 when compared with patients with sTG 5–20 ng/mL

## 4. Discussion

This study was based on data accumulated over two decades in a UK tertiary referral centre, and it demonstrates that up to a third of consecutive patients operated on for thyroid cancer achieve unmeasurable stimulated thyroglobulin (sTG) levels before receiving radioactive iodine ablation (RIA). Using an assay with a 0.2 ng/mL threshold as the detection rate, up to one in five patients would still be declared to have undetectable sTG before proceeding with RIA. Patients with unmeasurable/low sTG were more likely to have lower T-staging, but it was interesting to observe that the same outcome was obtained in up to a third of patients presenting with N1b disease, suggesting that the extent of disease at the initial diagnosis does not preclude achieving this favourable postoperative biochemical profile.

RIA was performed at a median of 12 weeks after thyroidectomy, and this timeline compares favourably with the practice reported from other centres. Furthermore, it has also been shown that the long-term outcome of early/low-risk thyroid cancers is not influenced by whether the RIA is administered early (median 3 months) or later (median 6 months) postoperatively [27]. The dose of 3.7 GBq I^131^ used routinely for RIA in this study was relatively high compared with current practice. A decade ago, a meta-analysis of 13 randomised control trials (RCTs) including 3352 patients found no statistical differences in successful ablation rates between a 100 mCi (3.7 GBq), 60 mCi, and 30 mCi (1.1 GBq) dose and failed to conclude which activity of I^131^ results in the most successful ablation rate [28]. Two subsequent prospective RCTs conducted in low- to intermediate-risk DTC patients showed that low-dose (1.1 GBq, 30 mCi) and high-dose (3.7 GBq, 100 mCi) of I^131^ ablation regimens had similar efficacies in eliminating normal thyroid residual tissue [29,30]. This led to a change in practice in recent years that was not captured by this study, as the recruitment stopped in 2019 in order to allow reasonable follow-up for all patients included in the analysis.

This study was focused on the subgroup of patients who achieved unmeasurable sTG levels following thyroid surgery before undergoing RIA. Measurement of serum TG levels is an important prognostic value in the management of DTC, as it is the most accurate means by which residual or recurrent DTC can be detected [24], but a threshold for pre-ablation sTG that impacts on outcomes is yet to be agreed upon (studies summarised in Table 4). 

Though not widely adopted, using sTG < 1 ng/mL after thyroidectomy as a criterion to spare low-risk patients from RIA has already been implemented in some centres. In a prospective study of 136 patients with PTC who underwent total thyroidectomy with apparently complete tumour resection, after a median follow-up of 44 months, 134 patients (98.5%) continued to have TG < 1 ng/mL during therapy with levothyroxine and had negative neck ultrasound scans. There was only one case of recurrence, even among the 60 patients with tumours >4 cm or minimal extrathyroidal invasion [40]. A further retrospective review of 278 patients with DTC showed that undetectable sTG combined with negative neck US have a 98% negative predictive value for disease-free survival [41]. Similar to these data, 94% of patients with sTG < 1 ng/mL remained with unmeasurable TG < 0.2 ng/mL during an average follow-up of over 7 years. Based on such data, the follow-up criteria for such patients should be revised, and the intensity of assessment should be ‘relaxed’ in future years.

The need to measure sTG during long-term follow-up is expected to decline as the availability of high-sensitivity TG assays increases [42,43,44]. These assays, which have functional sensitivities of <0.2 ng/mL, may allow earlier detection of ‘malignant’ TG production, even while patients are still taking levothyroxine. A study conducted in 2007 compared the diagnostic values of seven progressively sensitive TG assays [45]. A detection limit of 0.9 ng/mL was associated with a low sensitivity in identifying persistent disease (40%). Sensitivity doubled (to almost 80%) when the assays with the lowest detection limits (0.11 and 0.02 ng/mL) were tested, but the cost of this improvement was a considerable loss in specificity. Based on their data, the authors concluded that the best trade-off was a functional assay sensitivity between 0.2 and 0.3 ng/mL, which offers an improved diagnostic sensitivity without significantly diminishing the specificity. The data presented in this study was accumulated over a long time span that included a shift to such a high sensitivity assay, with a 0.2 ng/mL detection limit, and the results confirmed that patients whose pre-ablation sTG is <0.2 ng/mL have a very low incidence of negative clinical incidents (defined by recurrence or mortality).

An immunoassay (IMA) for detecting TG superseded a radioimmunoassay halfway through this study. Whilst most laboratories use IMA in preference to radioimmunoassays for its shorter incubation times [46,47] and increased sensitivity [48], IMA is prone to interference by endogenous thyroglobulin antibodies (TgAbs) which may underestimate serum TG concentrations. As TgAb is detected in approximately 20% of patients with DTC [49], this may pose a significant problem in the follow-up of DTC patients. It is recommended that TgAb be measured every time TG is assessed, preferably using an assay from the same manufacturer. However, there is so much heterogeneity across all TgAb assays, with mounting evidence demonstrating wide inter-assay variability [26,46,50], that even different laboratories using the same assay may produce considerably different results [48]. As there are no validated methods for completely eliminating the interference from TgAb, 49 patients with positive TgAb were included in this study.

Quantification of the cervical uptake after RIA can be used as a criterion for the completeness of surgical ablation of all thyroid tissue. These uptake figures provide valuable information, as up to 70% of thyroid cancers are confined to the thyroid bed at diagnosis, and the majority of patients with extrathyroidal disease have only regional lymph node involvement rather than widespread metastatic disease. Hence, I^131^ scintigraphy can quantify the degree of residual cervical thyroid uptake related to incomplete tumour resection [51,52]. The data presented in this analysis showed that patients with low sTG had significantly lower median percentage uptake, but there was significant overlap between subgroups (Figure 3).

It had previously been suggested that nearly 30% of thyroid cancers recur, even after 20–30 years of initial treatment [53]. As such, patients have been historically kept on hospital-based follow-up in dedicated outpatient clinics for 5 to 10 years following diagnosis and treatment due to the unvalidated assumption that this will be able to detect cancer recurrences early and, therefore, improve patient prognosis [54]. As the treatment of DTC becomes increasingly individually tailored according to patient and disease factors, it is anticipated that follow-up strategies will also reflect stratification based on risk. Future protocols based on the dynamic risk stratification in response to therapy should differentiate the majority of those with low-risk tumours that reach undetectable sTG (and for whom follow-up should be less intense) from those with recurrent or metastatic disease (on whom time and financial resources should be focused). This will provide maximum benefit for those with advanced disease while safely relaxing the follow-up of those with low-risk tumours. Data in support of such a protocol is increasingly convincing. For example, follow-up data from 1020 PTC patients showed that at 1 year after treatment, 948 patients had no structural/functional evidence of disease, and after a median follow-up of 10 years (range 5–20 years), only 1.4% of these patients had recurrence in cervical lymph nodes or the thyroid bed—all occurring within the first 8 years after treatment [55]. The current study brings further evidence that it would be safe to discharge patients with very low pre-ablation sTG from regular follow-up and that patient-initiated follow-up, similar to the system created for patients with other types of malignancy, should be encouraged.

This study is limited by the retrospective nature of the cohort identification. Furthermore, the long recruitment period (2001–2019) introduces significant heterogeneity in surgical indications, RIA protocols, TG assays, and follow-up strategies. None of these temporal confounders could have been statistically adjusted, and they are deemed unavoidable when collecting a large cohort of patients operated in a single centre. These problems could be addressed in a multi-centre prospective recruitment of patients, and the data presented in this paper could backup the rationale for such a study. The length of follow-up in this study (average 8 years) was relatively short; hence, we cannot comment on later rates of local recurrence or mortality.

## 5. Conclusions

This study demonstrated that over a third of consecutive unselected patients undergoing thyroid surgery for DTC achieved pre-ablation sTG of less than 5 ng/mL before undergoing RIA. The criteria used to identify patients with unmeasurable sTG differed throughout the study period according to the limit of detectability of the assay used, and the vast majority of recent patients assessed with high sensitivity assay of 0.2 ng/mL showed a very low risk of negative events during follow-up. Whether such patients could be streamlined into patient-initiated follow-up rather than be included in lengthy annual reviews needs to be explored in a prospective study.

## Figures and Tables

**Table 1:** Clinical and pathological details of patients stratified based on the value of pre-ablation stimulated thyroglobulin levels.

	Group A sTG Unmeasurable as <0.2 ng/mL	Group B sTG 0.2–5 ng/mL	Group C sTG Unmeasurable as <5 ng/mL	Group D sTG 5–20 ng/mL	Group E sTG > 20 ng/mL	TOTAL
Number	38	79	70	71	73	**331**
Gender (female, %)	34 (89%)	46 (58%)	54 (77%)	50 (62%)	46 (63%)	230 (70%)
Age	48.7 ± 15.5	48.0 ± 16.9	48.7 ± 14.3	50.5 ± 17.8	53.5 ± 20.5	
Pathology						
Histology type:						
Papillary (PTC)	31	60	56	53	52	252
Follicular (FTC)	7	19	14	18	21	79
T stage:						
Tx/unknown	0	9	7	15	3	34
T1	10	14	18	17	13	72
T2	12	19	35	17	13	96
T3	16	37	10	21	37	121
T4	0	0	0	1	7	8
N stage:						
Nx/unknown	21	43	40	52	35	191
N0	3	15	17	4	7	46
N1a	6	9	6	6	7	34
N1b	8	12	7	9	24	60
M stage:						
M0	38	76	69	71	66	320
M1	0	3	1	0	7	11
MACIS score						
Average	6.02 ± 1.25	5.79 ± 1.40	5.50 ± 1.07	6.14 ± 1.41	6.69 ± 1.84	
Score > 7	9/36 (25%)	14/70 (20%)	5/63 (8%)	17/54 (31%)	30/69 (43%)	
Surgery performed						
Lobectomy/completion thyroidectomy	16 (42%)	34 (43%)	31 (44%)	28 (39%)	19 (26%)	128
Total thyroidectomy	12 (32%)	33 (41%)	35 (50%)	34 (48%)	33 (45%)	147
Lateral neck lymph node dissection (synchronous)	10 (26%)	12 (15%)	4 (6%)	9 (12%)	21 (29%)	56
Operated in ‘central hub’	27 (71%)	52 (66%)	53 (76%)	42 (59%)	55 (75%)	229 (69%)

**Table 2:** Cervical uptake after radioactive iodine ablation.

	Median Value	IQR
Group A	0.39%	0.12–0.73%
Group B	1.04%	0.45–2.75%
Group C	1.0%	0.27–2.2%
Group D	1.9%	0.6–5.3%
Group E	2.%	0.53–3.85%

**Table 3:** Oncological outcomes in patients stratified based on the pre-ablation stimulated thyroglobulin levels.

	Group A sTG < 0.2 ng/mL	Group B sTG 0.2–5 ng/mL	Group C sTG Unmeasurable as <5 ng/mL	Group D sTG 5–20 ng/mL	Group E sTG >20 ng/mL	Total
number	38	79	70	71	73	331
Length of follow-up (months)	92 ± 31	73 ± 29	128 ± 37	120 ± 54	90 ± 55	100 ± 48
Status at last follow-up						
No known disease	36	70	56	50	16	228
Biochemical recurrence	1	6	4	12	22	45
Radiological recurrence	0	0	2	1	9	12
Deceased with/without evidence metastatic disease	0/1	½	2/6	2/6	21/3	26/18

**Table 4:** Studies investigating cut-off points for stimulated thyroglobulin levels that impact on outcomes.

Ref.	Number of Patients	Cut-Off Point for sTG	Findings
[31]	502	2 ng/mL	Structural recurrence rate of up to 5% was seen in groups with sTG < 2 ng/mL vs. 30% in those with sTG ≥ 2 ng/mL
[32]	62	3.15 ng/mL	Highest sensitivity and specificity on ROC curve analysis
[22]	375	4.4 ng/mL	An optimal cut-off point of 4.4 ng/mL has sensitivity 72%; specificity of 72%, positive predictive value of 56%, and negative predictive value of 84% for incomplete response to treatment
[33]	960	7.5 ng/mL	Patients with sTG > 7.5 ng/mL before the first ^131^I therapy were more likely to have a poor long-term efficacy after full ^131^I therapy
[34]	166	7.55 ng/mL	sTG < 7.55 ng/mL was associated with excellent response (RR 2.17, 95% CI 1.52–3.10, *p* < 0.001)
[24]	3497 (meta-analysis)	10 ng/mL	Patients with postoperative pre-ablation sTG < 10 ng/mL have only 6% risk of persistent disease
[35]	126 (children)	13.75 ng/mL	sTG > 13.75 ng/mL was the most powerful predictor of first-year incomplete response
[36]	130	20.2 ng/mL	sTG > 20.2 ng/mL is an independent risk factor for persistent/recurrent disease (odds ratio = 5.6; *p* < 0.001)
[37]	452	26.75 ng/mL	Differentiating structural incomplete response from either excellent, indeterminate, or biomedical incomplete responses
[38]	82	30 ng/mL	When sTG was > 30 ng/mL, the risk of relapse increased six-fold
[39]	126 (children)	112.4 ng/mL	Pre-ablation sTG > 112.4 ng/mL was significantly associated with non-remission [39]

## Data Availability

Data supporting reported results is stored in anonymised forms by the authors.

## Abbreviations

The following abbreviations are used in this manuscript:

sTG	Stimulated thyroglobulin
RIA	Radioactive iodine ablation

## References

[1] Rahib L., Smith B.D., Aizenberg R., Rosenzweig A.B., Fleshman J.M., Matrisian L.M. (2014). Projecting cancer incidence and deaths to 2030: The unexpected burden of thyroid, liver, and pancreas cancers in the United States. Cancer Res..

[2] Davies L., Welch H.G. (2014). Current thyroid cancer trends in the United States. JAMA Otolaryngol. Head Neck Surg..

[3] McCarthy M. (2014). US thyroid cancer rates are epidemic of diagnosis not disease, study says. BMJ.

[4] Brito J.P., Morris J.C., Montori V.M. (2013). Thyroid cancer: Zealous imaging has increased detection and treatment of low risk tumours. BMJ.

[5] Vollmer R.T. (2014). Revisiting overdiagnosis and fatality in thyroid cancer. Am. J. Clin. Pathol..

[6] Siegel R., Ma J., Zou Z., Jemal A. (2014). Cancer statistics, 2014. CA Cancer J. Clin..

[7] Davies L., Hoang J.K. (2021). Thyroid cancer in the USA: Current trends and outstanding questions. Lancet Diabetes Endocrinol..

[8] Davies L., Welch H.G. (2006). Increasing incidence of thyroid cancer in the United States, 1973–2002. JAMA.

[9] Welch H.G., Black W.C. (2010). Overdiagnosis in cancer. J. Natl. Cancer Inst..

[10] Haugen B.R., Alexander E.K., Bible K.C., Doherty G.M., Mandel S.J., Nikiforov Y.E., Pacini F., Randolph G.W., Sawka A.M., Schlumberger M. (2016). 2015 American Thyroid Association Management Guidelines for Adult Patients with Thyroid Nodules and Differentiated Thyroid Cancer: The American Thyroid Association Guidelines Task Force on Thyroid Nodules and Differentiated Thyroid Cancer. Thyroid.

[11] Schlumberger M.J. (1998). Papillary and follicular thyroid carcinoma. N. Engl. J. Med..

[12] Pelttari H., Välimäki M.J., Löyttyniemi E., Schalin-Jäntti C. (2010). Post-ablative serum thyroglobulin is an independent predictor of recurrence in low-risk differentiated thyroid carcinoma: A 16-year follow-up study. Eur. J. Endocrinol..

[13] Dretzke J., Lorenc A., Adriano A., Herd C., Mehanna H., Nankivell P., Moore D.J., The PETNECK2 Research Team (2023). Systematic review of patients’ and healthcare professionals’ views on patient-initiated follow-up in treated cancer patients. Cancer Med..

[14] Hoeg B.L., Bidstrup P.E., Karlsen R.V., Friberg A.S., Albieri V., Dalton S.O., Saltbaek L., Andersen K.K., Horsboel T.A., Johansen C. (2019). Follow-up strategies following completion of primary cancer treatment in adult cancer survivors. Cochrane Database Syst. Rev..

[15] Patel H., Drinkwater K., Stewart A. (2024). National Survey of Current Follow-up Protocols for Patients Treated for Endometrial Cancer in the UK. Clin. Oncol..

[16] Giovanella L., D’aurizio F., Ovčariček P.P., Görges R. (2024). Diagnostic, Theranostic and Prognostic Value of Thyroglobulin in Thyroid Cancer. J. Clin. Med..

[17] Harvey R.D., Matheson N.A., Grabowski P.S., Rodger A.B. (1990). Measurement of serum thyroglobulin is of value in detecting tumour recurrence following treatment of differentiated thyroid carcinoma by lobectomy. Br. J. Surg..

[18] Heemstra K.A., Liu Y.Y., Stokkel M., Kievit J., Corssmit E., Pereira A.M., Romijn J.A., Smit J.W.A. (2007). Serum thyroglobulin concentrations predict disease-free remission and death in differentiated thyroid carcinoma. Clin. Endocrinol..

[19] Lin J., Huang M., Hsu B.R., Chao T., Hsueh C., Liu F., Liou M., Weng H. (2002). Significance of postoperative serum thyroglobulin levels in patients with papillary and follicular thyroid carcinomas. J. Surg. Oncol..

[20] Rosario P.W., Xavier A.C.M., Calsolari M.R. (2011). Value of Postoperative Thyroglobulin and Ultrasonography for the Indication of Ablation and ^131^I Activity in Patients with Thyroid Cancer and Low Risk of Recurrence. Thyroid.

[21] Spaas M., Decallonne B., Laenen A., Billen J., Nuyts S. (2018). Prognostic Value of Stimulated Thyroglobulin Levels at the Time of Radioiodine Administration in Differentiated Thyroid Cancer. Eur. Thyroid. J..

[22] Nóbrega G., Cavalcanti M., Leite V., Vilar L., Brandão S.C.S. (2022). Value of stimulated pre-ablation thyroglobulin as a prognostic marker in patients with differentiated thyroid carcinoma treated with radioiodine. Endocrine.

[23] Tian T., Xu Y., Zhang X., Liu B. (2021). Prognostic Implications of Preablation Stimulated Tg: A Retrospective Analysis of 2500 Thyroid Cancer Patients. J. Clin. Endocrinol. Metab..

[24] Webb R.C., Howard R.S., Stojadinovic A., Gaitonde D.Y., Wallace M.K., Ahmed J., Burch H.B. (2012). The Utility of Serum Thyroglobulin Measurement at the Time of Remnant Ablation for Predicting Disease-Free Status in Patients with Differentiated Thyroid Cancer: A Meta-Analysis Involving 3947 Patients. J. Clin. Endocrinol. Metab..

[25] Rosario P.W., Furtado M.d.S., Mourão G.F., Calsolari M.R. (2015). Patients with Papillary Thyroid Carcinoma at Intermediate Risk of Recurrence According to American Thyroid Association Criteria Can Be Reclassified as Low Risk When the Postoperative Thyroglobulin Is Low. Thyroid.

[26] Clark P., Franklyn J. (2012). Can we interpret serum thyroglobulin results?. Ann. Clin. Biochem. Int. J. Biochem. Lab. Med..

[27] Tsirona S., Vlassopoulou V., Tzanela M., Rondogianni P., Ioannidis G., Vassilopoulos C., Botoula E., Trivizas P., Datseris I., Tsagarakis S. (2014). Impact of early vs. late postoperative radioiodine remnant ablation on final outcome in patients with low-risk well-differentiated thyroid cancer. Clin. Endocrinol..

[28] Fang Y., Ding Y., Guo Q., Xing J., Long Y., Zong Z. (2013). Radioiodine therapy for patients with differentiated thyroid cancer after thyroidectomy: Direct comparison and network meta-analyses. J. Endocrinol. Investig..

[29] Mallick U., Harmer C., Yap B., Wadsley J., Clarke S., Moss L., Nicol A., Clark P.M., Farnell K., McCready R. (2012). Ablation with low-dose radioiodine and thyrotropin alfa in thyroid cancer. N. Engl. J. Med..

[30] Schlumberger M., Catargi B., Borget I., Deandreis D., Zerdoud S., Bridji B., Bardet S., Leenhardt L., Bastie D., Schvartz C. (2012). Tumeurs de la Thyroide Refractaires Network for the Essai Stimulation Ablation Equivalence T. Strategies of radioiodine ablation in patients with low-risk thyroid cancer. N. Engl. J. Med..

[31] Jayasekara J., Jonker P., Lin J.F., Engelsman A.F., Wong M.S., Kruijff S., Aniss A., Learoyd D., Bligh R.C., Glover A. (2020). Early postoperative stimulated serum thyroglobulin quantifies risk of recurrence in papillary thyroid cancer. Surgery.

[32] Elmaraghi C., Shaaban M., Reda C. (2022). Prognostic value of postoperative stimulated thyroglobulin in differentiated thyroid cancer. Ann. Endocrinol..

[33] Yin X., Lu C., Sun D., Ji Y., Wang Y., Zheng H., Ma Z., Jia Q., Tan J., Zheng W. (2024). Stimulating thyroglobulin to TSH ratio predict long-term efficacy of 131I therapy in patients with differentiated thyroid cancer after total thyroidectomy: A retrospective study. Endocrine.

[34] Jaeger F., Eidt L.B., Guidolin K., Landenberger G.M.C., Bündchen C., Golbert L., Mattevi V.S., Meyer E.L.d.S. (2024). Is Stimulated Thyroglobulin Before Radioiodine Therapy a Useful Tool in Predicting Response to Initial Therapy in Patients with Differentiated Thyroid Carcinoma?. Horm. Metab. Res..

[35] Nesari Javan F., Askari E., Shafiei S., Roshanravan V., Aghaei A., Ayati N., Zakavi S.R. (2024). The Prognostic Power of Preablation Stimulated Thyroglobulin in Children with Differentiated Thyroid Cancer. Endocr. Pract..

[36] Wang Y., Wu J., Jiang L., Zhang X., Liu B. (2022). Prognostic value of post-ablation stimulated thyroglobulin in differentiated thyroid cancer patients with biochemical incomplete response: A bi-center observational study. Endocrine.

[37] Yang X., Liang J., Li T., Zhao T., Lin Y. (2016). Preablative Stimulated Thyroglobulin Correlates to New Therapy Response System in Differentiated Thyroid Cancer. J. Clin. Endocrinol. Metab..

[38] Krajewska J., Jarzab M., Czarniecka A., Roskosz J., Kukulska A., Handkiewicz-Junak D., Puch Z., Wygoda Z., Paliczka-Cieslik E., Kropinska A. (2016). Ongoing risk stratification for differentiated thyroid cancer (DTC)-stimulated serum thyroglobulin (Tg) before radioiodine (RAI) ablation, the most potent risk factor of cancer recurrence in M0 patients. Endokrynol. Pol..

[39] Wang C., Li Y., Zhang Y., Wang G., Liu X., Zhang Y., Wang Z., Si Z., Li F., Lu G. (2024). Prognostic value of pre-ablation stimulated thyroglobulin in children and adolescents with differentiated thyroid cancer. Future Oncol..

[40] Rosario P.W., Mineiro Filho A.F., Prates B.S., Silva L.C., Calsolari M.R. (2012). Postoperative stimulated thyroglobulin of less than 1 ng/ml as a criterion to spare low-risk patients with papillary thyroid cancer from radioactive iodine ablation. Thyroid.

[41] Klubo-Gwiezdzinska J., Burman K.D., Van Nostrand D., Wartofsky L. (2011). Does an undetectable rhTSH-stimulated Tg level 12 months after initial treatment of thyroid cancer indicate remission?. Clin. Endocrinol..

[42] Smallridge R.C., Meek S.E., Morgan M.A., Gates G.S., Fox T.P., Grebe S., Fatourechi V. (2007). Monitoring thyroglobulin in a sensitive immunoassay has comparable sensitivity to recombinant human TSH-stimulated thyroglobulin in follow-up of thyroid cancer patients. J. Clin. Endocrinol. Metab..

[43] Malandrino P., Latina A., Marescalco S., Spadaro A., Regalbuto C., Fulco R.A., Scollo C., Vigneri R., Pellegriti G. (2011). Risk-Adapted management of differentiated thyroid cancer assessed by a sensitive measurement of basal serum thyroglobulin. J. Clin. Endocrinol. Metab..

[44] Castagna M.G., Maino F., Cipri C., Belardini V., Theodoropoulou A., Cevenini G., Pacini F. (2011). Delayed risk stratification, to include the response to initial treatment (surgery and radioiodine ablation), has better outcome predictivity in differentiated thyroid cancer patients. Eur. J. Endocrinol..

[45] Schlumberger M., Hitzel A., Toubert M.E., Corone C., Troalen F., Schlageter M.H., Claustrat F., Koscielny S., Taieb D., Toubeau M. (2007). Comparison of seven serum thyroglobulin assays in the follow-up of papillary and follicular thyroid cancer patients. J. Clin. Endocrinol. Metab..

[46] Spencer C.A., Bergoglio L.M., Kazarosyan M., Fatemi S., LoPresti J.S. (2005). Clinical Impact of Thyroglobulin (Tg) and Tg autoantibody method differences on the management of patients with differentiated thyroid carcinomas. J. Clin. Endocrinol. Metab..

[47] Spencer C.A., Takeuchi M., Kazarosyan M. (1996). Current status and performance goals for serum thyroglobulin assays. Clin. Chem..

[48] Verburg F.A., Luster M., Cupini C., Chiovato L., Duntas L., Elisei R., Feldt-Rasmussen U., Rimmele H., Seregni E., Smit J.W. (2013). Implications of thyroglobulin antibody positivity in patients with differentiated thyroid cancer: a clinical position statement. Thyroid.

[49] Spencer C.A., Lopresti J.S. (2008). Measuring thyroglobulin and thyroglobulin autoantibody in patients with differentiated thyroid cancer. Nat. Clin. Pract. Endocrinol. Metab..

[50] Spencer C., Petrovic I., Fatemi S. (2011). Current thyroglobulin autoantibody (TgAb) assays often fail to detect interfering TgAb that can result in the reporting of falsely low/undetectable serum Tg IMA values for patients with differentiated thyroid cancer. J. Clin. Endocrinol. Metab..

[51] Cailleux A.F., Baudin E., Travagli J.P., Ricard M., Schlumberger M. (2000). Is Diagnostic iodine-131 scanning useful after total thyroid ablation for differentiated thyroid cancer?. J. Clin. Endocrinol. Metab..

[52] Torlontano M., Crocetti U., D’Aloiso L., Bonfitto N., Di Giorgio A., Modoni S., Valle G., Frusciante V., Bisceglia M., Filetti S. (2003). Serum thyroglobulin and 131I whole body scan after recombinant human TSH stimulation in the follow-up of low-risk patients with differentiated thyroid cancer. Eur. J. Endocrinol..

[53] Tuttle R.M., Leboeuf R. (2008). Follow up approaches in thyroid cancer: A risk adapted paradigm. Endocrinol. Metab. Clin. N. Am..

[54] Newton C., Nordin A., Rolland P., Ind T., Larsen-Disney P., Martin-Hirsch P., Beaver K., Bolton H., Peevor R., Fernandes A. (2020). British Gynaecological Cancer Society recommendations and guidance on patient-initiated follow-up (PIFU). Int. J. Gynecol. Cancer.

[55] Durante C., Montesano T., Torlontano M., Attard M., Monzani F., Tumino S., Costante G., Meringolo D., Bruno R., Trulli F. (2013). Papillary thyroid cancer: Time course of recurrences during postsurgery surveillance. J. Clin. Endocrinol. Metab..

